# Unraveling the fatigue puzzle: insights into the pathogenesis and management of IBD-related fatigue including the role of the gut-brain axis

**DOI:** 10.3389/fmed.2024.1424926

**Published:** 2024-07-03

**Authors:** Marie Truyens, Hannah Lernout, Martine De Vos, Debby Laukens, Triana Lobaton

**Affiliations:** ^1^Department of Internal Medicine and Pediatrics, Ghent University, Ghent, Belgium; ^2^Department of Gastroenterology, University Hospital Ghent, Ghent, Belgium; ^3^VIB Center for Inflammation Research (IRC), Ghent University, Ghent, Belgium; ^4^Ghent Gut Inflammation Group (GGIG), Ghent University, Ghent, Belgium

**Keywords:** fatigue, Crohn’s disease, ulcerative colitis, tiredness, gut-brain axis

## Abstract

A significant percentage of patients with an inflammatory bowel disease (IBD) encounter fatigue which can profoundly diminish patients’ quality of life, particularly during periods of disease remission when gastrointestinal symptoms have receded. Various contributing risk factors have been identified including active inflammation, anemia, psychological, lifestyle and drug-related factors. While addressing these risk factors has been suggested as the initial approach to managing fatigue, a considerable number of patients still experience persisting symptoms, the primary causes of which remain incompletely understood. Recent insights suggest that dysfunction of the gut-brain axis may play a pathogenic role. This review provides an overview of established risk factors for fatigue, alongside emerging perspectives on the role of the gut-brain axis, and potential treatment strategies.

## 1 Introduction

In recent years, there have been significant advances in the understanding of inflammatory pathways involved in inflammatory bowel diseases (IBD), leading to a steep increase in the number of available treatments for IBD (1). With the expanding treatment options and advancements in diagnostic tools, the aspirations in treatment goals have shifted and a treat to target strategy is proposed, which includes long-term treatment goals such as a normalized quality of life (QoL) (2). The latter has gained more importance in recent decades, since it has become clear that IBD symptoms are not limited to the gastrointestinal tract. Patients with IBD often encounter disabling fatigue which can severely influence their social and interpersonal functioning and significantly reduce their QoL (3). During stages of active disease up to 72% of patients with IBD report fatigue, but even when disease remission is reached, and intestinal symptoms become less dominant over daily life, up to 42% of patients remain fatigued (4). This prevalence is remarkably higher than the 7% rate of fatigue observed in the general population (4, 5).

Psychological comorbidities of IBD not only reduce QoL (6, 7), but they can also impact the course of IBD itself, implying bidirectional gut-brain interactions in IBD. On the one hand, active IBD is associated with higher rates of psychological symptoms (4, 8). On the other hand, under-recognized and/or suboptimal treated mental health problems are associated with worse IBD-related outcomes including an increased risk of flare-ups, hospitalizations, surgery and corticosteroid use (9–13). The incidence of IBD is the highest in the 15–29-year-old age group (14), and impairment due to IBD has been shown to affect educational and employment prospects, implying an increased socioeconomic burden (15, 16). Moreover, fatigue in patients with IBD is associated with adverse outcomes and increased IBD-related and all-cause healthcare costs (13).

This review covers the definition and assessment of IBD-related fatigue, followed by an account of factors contributing to this condition. Next, an overview on recent insights into the pathophysiology will be provided, with an emphasis on the gut-brain axis. Finally, potential management strategies are discussed.

## 2 Definition of IBD-related fatigue and its assessment

Despite the high prevalence of fatigue, there is still a lack of clarity in the terminology, definition, and conceptualization of IBD-related fatigue, with different definitions being used throughout the current literature (17). Fatigue can be defined as an ongoing exhaustion or tiredness that is disproportionate or unrelated to activity or exertion and not alleviated by rest (18, 19). Several authors have suggested a multidimensional concept of fatigue including a physical, cognitive, and affective dimension (17, 20). The physical aspect of fatigue consists of the subjective feeling of weakness and the objective measurable decrease in physical activity and/or performance (20). Secondly, the cognitive aspect can manifest itself as a subjective difficulty in concentrating and the objective decrease in cognitive function or performance. Lastly, the affective component includes a decrease in motivation and mood (20).

The same unclarity that exists in the definition of IBD-related fatigue continues in the lack of consensus on how to assess fatigue (21). Over 250 different scales have been reported, of which at least nine have been used for research on IBD-related fatigue and five have been validated for this purpose. These include the functional assessment of chronic illness therapy fatigue (FACIT-F), the fatigue questionnaire (FQ), multidimensional assessment fatigue (MAF), the multidimensional fatigue inventory (MFI), and the inflammatory bowel disease fatigue (IBD-F); of which the latter is recommended for research and clinical use (22, 23).

## 3 Factors associated with IBD-related fatigue

### 3.1 Active disease correlates strongly with fatigue burden

Disease activity influences fatigue through various mechanisms. Activation of the systemic immune system increases central cytokine release, inducing sickness behavior similar to SARS-CoV-2 infection (24, 25). Additionally, the symptom burden, including frequent stools and nocturnal diarrhea, reduces energy levels (26). The important role of disease activity is clearly reflected by the significant difference in fatigue prevalence in patients with active disease compared to those in remission (4) and by the similar fatigue rates in other immune mediated inflammatory disorders (IMIDs) (27, 28). In multiple IBD-studies, clinical disease activity scores have been linked to increased fatigue burden (26, 29–31) and long-term persistence of disease activity has been associated with higher fatigue levels (32). The same trend was seen when assessing the correlation between fatigue and biochemical indices of inflammation such as C-reactive protein (CRP) (31, 33), erythrocyte sedimentation rate (34) and fecal calprotectin (35). Up to date, endoscopic disease activity has only been linked to fatigue in patients with ulcerative colitis (UC) (35, 36).

**Figure 1:**
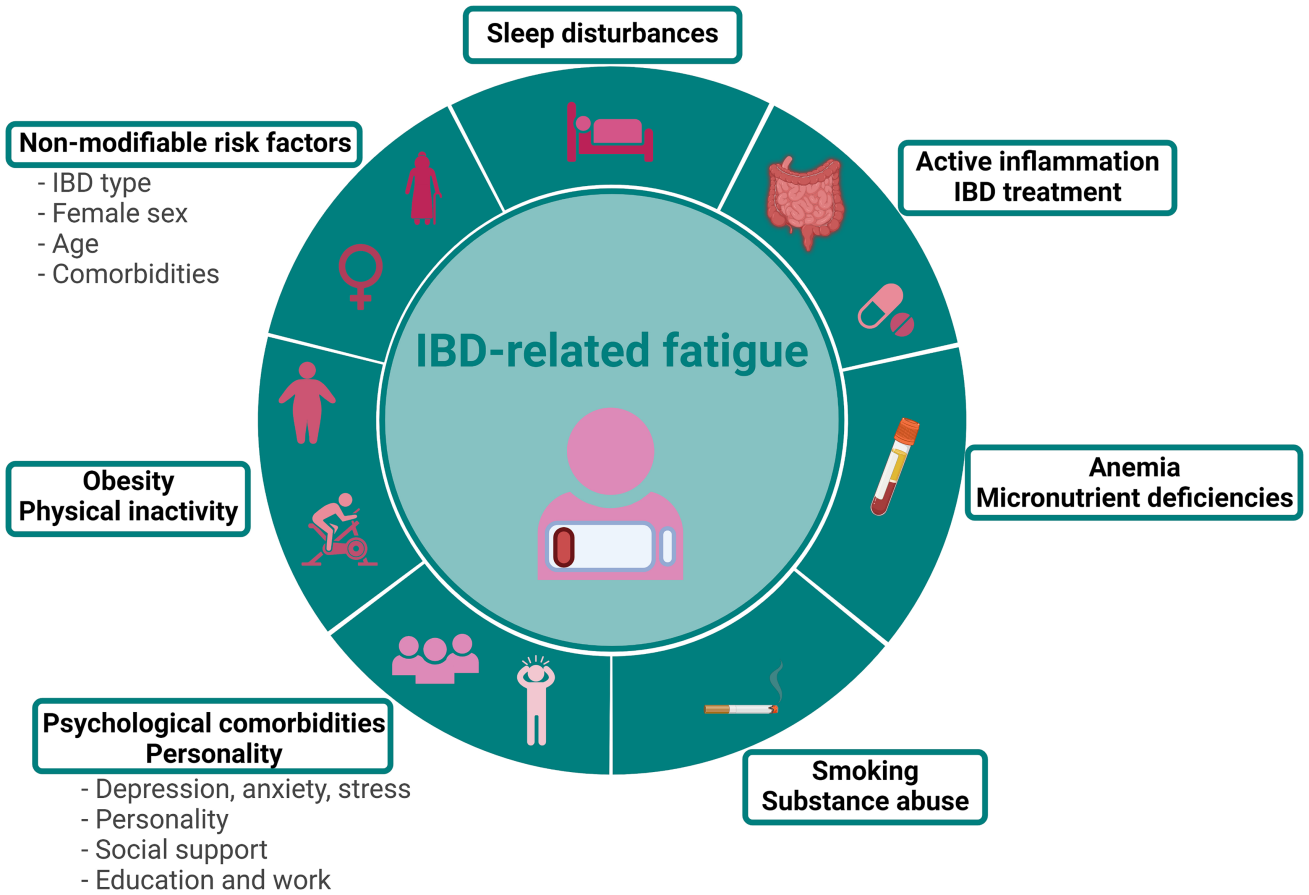
Risk factors associated with IBD-related fatigue. Figure created with BioRender.com.

The relationship between fatigue and IBD-treatments is rather complex. Fatigue is a reported side effect of treatments such as immunomodulators and TNF-α inhibitors (26, 37–41). However, the necessity for these treatments suggests more severe disease, indicating that disease severity may have a greater impact on fatigue than the medication itself (26). Continued use of TNF-α inhibitors for 1 year has been associated with lower fatigue levels, likely due to achieving disease remission (41). Even though an improvement of disease activity does parallel amelioration of fatigue, fatigue often persists in patients in remission (30, 42–45), indicating that disease activity is not the sole contributor to fatigue (Figure 1).

### 3.2 The persistence of fatigue in patients with IBD in remission: a role for subclinical inflammation?

Typically, “sickness behavior” subsides once the initial acute illness ends. However, prolonged cytokine activation and dysregulation have been identified in chronic conditions such as long COVID, which can be associated with severe fatigue (25). When assessing cytokine patterns in fatigued patients with IBD in remission, conflicting results have been reported. In the study of Vogelaar et al., fatigue was associated with increased TNF-α and IFN-γ levels, and the immune profiles of these patients suggested a chronically active and T-helper cell 1 (T_h_1)-skewed immune system, despite clinical disease remission (46). Nevertheless, several other studies found no relationship between interleukin levels and fatigue in quiescent IBD (35, 42, 43). Thus, at present, insufficient evidence exists for a role of subclinical inflammation in fatigue pathogenesis during disease remission.

### 3.3 Micronutrient deficiencies and anemia

Anemia is a frequent complication of IBD and is mainly caused by chronic iron deficiency, anemia of chronic disease or a combination of both (47). In general, fatigue has been described as a symptom of anemia and this relationship was confirmed in several (18, 48) but not all studies (49–51). Iron plays a critical role in normal brain function and iron deficiency can negatively impact behavioral and mental health (52). Whereas some studies identified iron deficiency (in the absence of anemia) as a risk factor for fatigue (35, 53), this effect was not uniformly reported (30, 50, 51, 54).

Besides iron deficiency, patients with IBD are also at risk for deficiencies in other micronutrients but the role of these deficiencies in IBD-related fatigue is even more equivocal. Vitamin D deficiency is highly prevalent in IBD, ranging from 22 to 100% (55–57) and lower vitamin D levels has been linked to IBD-related fatigue (31) and muscle fatigue in patients with Crohn’s disease (CD) (58). Patients with IBD are also at higher risk for deficiencies in thiamine, vitamin B_12_ and folate, which in theory could lead to fatigue (59, 60) but studies supporting this association are lacking.

### 3.4 Psychological comorbidities and personality

Patients with IBD not only struggle with fatigue but also report high levels of depression (25%) and anxiety (35%), both of which are major risk factors for fatigue during active disease and remission (4, 8, 61, 62). Additionally, up to 51% of patients with IBD experience significant life stress (63), further increasing fatigue (30). Chronic diseases such as IBD can be associated with a significant burden on patients and not all patients have the same ability to cope with the disease and its consequences (64). Patients who experience increased disease-related worrying, negative perceptions about fatigue, feel a lack of control over their symptoms or engage in maladaptive behavioral coping strategies will experience higher levels of fatigue (65, 66). In addition, fatigue interference with daily life was negatively associated with a person’s sense of coherence, which can be defined as someone’s capacity to respond to stressful situations (64). On the other hand, patients with a self-directness and persistence dimension of personality experience lower fatigue levels (67). Stronger social support is associated with fewer psychological symptoms, yet nearly 70% of IBD patients face social constraints, and their fatigue burden is often underestimated by family and friends (68–70). Lower education levels (26), unemployment (40) and lower income are also significantly associated with higher fatigue and its interference with daily life (71).

### 3.5 Sleep disturbances

Up to 82% of patients with active IBD and 50% with inactive disease report disrupted sleep, compared to 32% of the general population (30). There is a bidirectional relationship between sleep and IBD: active disease leads to worse sleep quality due to higher symptom burden (72), and sleep disturbances in patients with CD in remission increase the risk of future flares (30, 72–74). Poor sleep quality is linked to increased fatigue, with associations noted with daytime sleepiness, prolonged sleep latency, sleep disturbances, and insomnia (67, 75). Furthermore, a recent study identified disturbed sleep in non-fatigued patients as a strong predictor of fatigue 6 months later (76). Importantly, sleep disturbances are also associated with other risk factors for fatigue such as disease activity and mood disorders and poor sleep quality is negatively correlated with vigorous physical activity, highlighting the complex interplay in fatigue etiology (72, 77).

### 3.6 Sarcopenia and obesity

High rates of sarcopenia are seen in patients with IBD (78), which has been related to severe fatigue (33). On the other hand, there is an ongoing global obesity epidemic, and up to 15–40% of adult patients with IBD are obese (79). Patients with obesity are not only less likely to reach clinical remission, but they also score worse on domains such as anxiety, depression, and fatigue (40, 79–81). Furthermore, individuals who follow an unhealthy diet, characterized by frequent consumption of foods like fatty red meat, sugary beverages, and fried dishes, reported heightened levels of fatigue (82).

### 3.7 Physical activity

Physical activity is increasingly being recognized for its beneficial effects on overall health, well-being, and QoL of patients with IBD (83). Conversely, nearly 30% of patients with non-severe IBD expressed concerns that physical activity might reactivate or exacerbate their disease status, a belief strongly linked to decreased physical activity levels (84). Patients with IBD are often more physically inactive (85–87), have higher rates of muscle fatigue (58), reduced hand grip strength (88) and lower quadriceps strength (86) as compared to their healthy peers. Importantly, patients feel that their disease imposes barriers on regular exercise, in part due to abdominal pain and urge, but mainly due to fatigue (89, 90). Patients with IBD-related fatigue showed significantly lower intensity of daily physical activity and reduced cardiorespiratory fitness (26, 91) and hand grip strength correlated significantly with fatigue levels (92). Both during active and quiescent stages of IBD, fatigue can restrict exercise capability, initiating a vicious cycle of physical inactivity, impaired physical fitness and worsening fatigue (91, 93).

### 3.8 Smoking and substance abuse

Nicotine use significantly impacts the risk of developing IBD and the disease course (94), and certain studies have linked smoking to higher rates of fatigue (31, 80). In addition to smoking, the use of psychotropics and narcotics is also higher in patients with IBD reporting fatigue compared to those that did not (74).

### 3.9 Non-modifiable risk factors

#### 3.9.1 IBD type and disease duration

Differences in fatigue levels exist between IBD entities, with a recent meta-analysis reporting a general fatigue prevalence of 51% in CD and 45% in UC (95). This may be attributed to variations in disease behavior and severity between the two entities. For instance, factors indicating more complicated disease such as penetrating phenotype, previous biological use, and intestinal surgery have also been associated with higher fatigue burden (26, 31, 96). Additionally, malnutrition and sarcopenia rates are higher in CD compared to UC which could also contribute to fatigue (78).

The association between disease duration and fatigue remains ambiguous. While one study identified disease chronicity as a factor contributing to fatigue (31) another study observed lower fatigue levels in patients with longer disease duration (97). Finally, one study showed no significant relationship between disease duration and fatigue (44).

#### 3.9.2 Sex and age

Several studies have identified a connection between the female sex and IBD-related fatigue, yet the reasons for the higher rates of fatigue in women remain uncertain (32, 35, 40, 42, 80, 98). Possibly, women do experience more severe fatigue compared to men, and additionally, women may have a greater willingness to report their (mental) health problems (71). Differential hormonal responses and their effects on the immune function might also explain part of the sex differences in fatigue (98).

In general, increasing age has been associated with lower levels of fatigue as compared to younger patients with IBD, potentially due to differences in coping strategies, responsibilities or adaptation (54, 97, 99, 100).

#### 3.9.3 Comorbidities of persistent fatigue

Up to 35% of patients with IBD in remission have symptoms compatible with irritable bowel syndrome (IBS) which can lead to a higher symptom burden, increased (unjustified) disease-related worrying and reduced QoL (66, 101, 102). Moreover, several studies have confirmed the important correlation between IBS and IBD-related fatigue (66, 99, 103).

Fatigue can be associated with endocrinopathies such as adrenal insufficiency, hypopituitarism, hypogonadism, and thyroid diseases and patients with IBD are known to be at risk for autoimmune disorders (104, 105). Patients with IBD also have a higher risk of EIMs that can involve several organ systems such as skin, joints, hepatobiliary tract, or eyes (106). The presence of an EIM correlated significantly with the severity of fatigue (49) and patients with fatigue had a higher prevalence of EIMs compared to patients without fatigue (26). Joint EIMs are the most common in IBD, and in the subanalysis, these were the EIMs significantly linked to fatigue (49).

Patients with IBD are at higher risk for infections, especially during immunosuppressive treatment (107). While some infections may manifest with tiredness, evidence supporting their role in fatigue pathogenesis is limited. Recently, systemic antibody repertoires were assessed in patients with quiescent IBD, and increased antibody responses toward viral (including Epstein–Barr virus) and bacterial antigens were seen in patients with higher fatigue levels (108). Moreover, in patients with an IMID a history of SARS-CoV-2 infection was associated with elevated fatigue levels (109).

Even though IBD is typically diagnosed in patients aged 20–40 years, a second rise in incidence is reported in 60–70-year-old patients (110). Additionally, as the population exhibits aging globally, an increase in IBD among elderly patients is expected (110). This leads to higher rates of comorbidities and polypharmacy, both of which have been linked with fatigue (111). Moreover, aging is associated with longer exposure to chronic inflammation and/or immunosuppression, increasing the risk of cancer development, again potentially contributing to fatigue (112). Thus, screening for endocrinopathies or other comorbidities can be advised in patients with IBD presenting with fatigue.

## 4 New insights into the pathophysiology of IBD-related fatigue: the gut-brain axis

**Figure 2:**
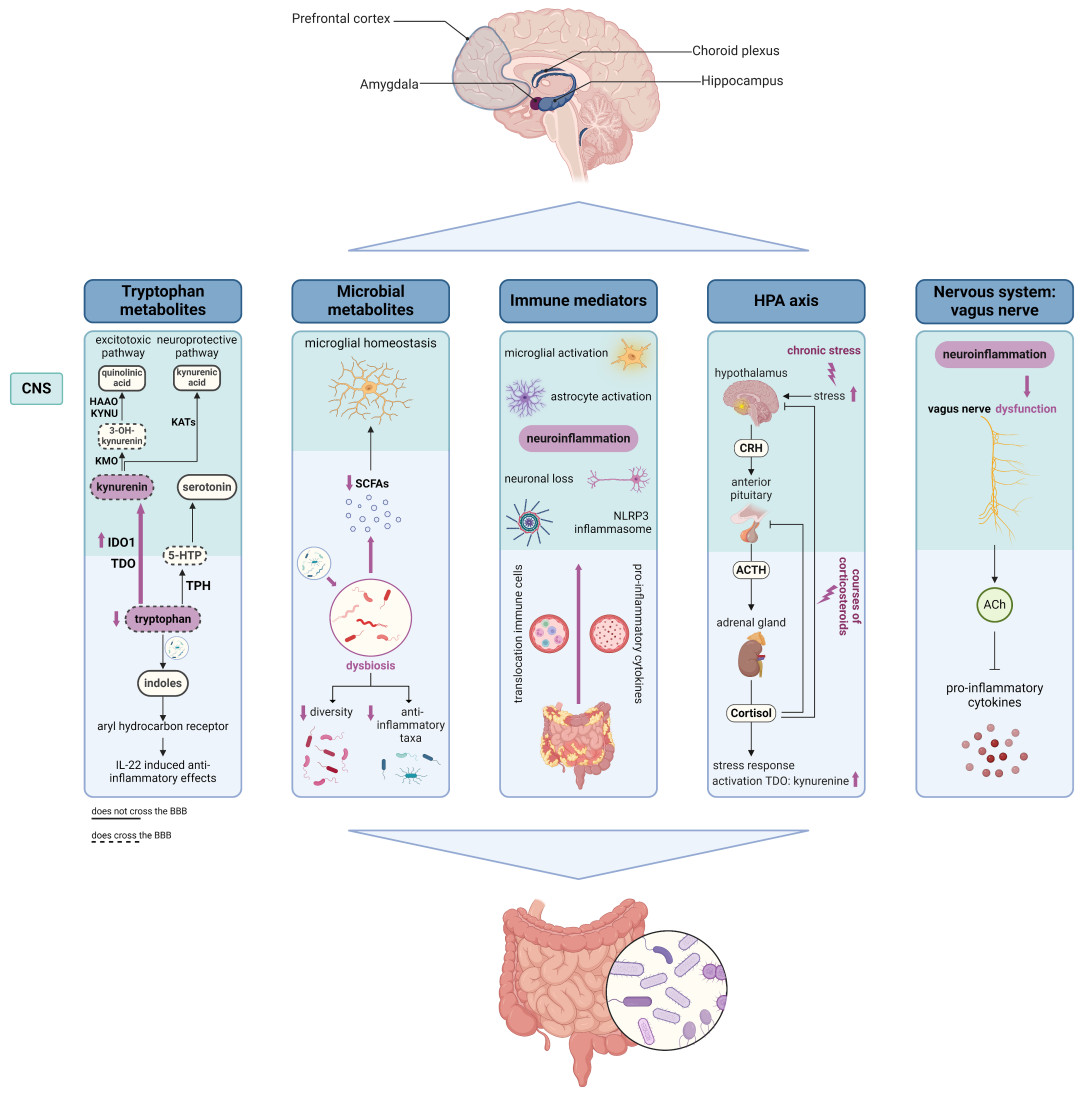
Schematic overview of different gut-brain communication pathways that could contribute to the pathogenesis of IBD-related fatigue. Figure created with BioRender.com; the HPA axis and potential impacts of IBD on the axis was created based on the Biorender.com template from Camilla Maria Fontana, University of Padova. 5-HTP, 5-hydroxytrypthophan; ACh, acetylcholine; ACTH, adrenocorticotropic hormone; BBB, blood–brain barrier; CNS, central nervous system; CRH, corticotropin releasing hormone; IBD, inflammatory bowel disease; HAAO, 3-hydroxyanthranillic acid 3,4-dioxygenase; KATs, kynurenine aminotransferases; KMO, kynurenine 3-monooxygenase; KYNU, kynureninase; IDO1, indoleamine 2,3-dioxygenase 1; SCFAs, short chain fatty-acids; TDO, tryptophan 2,3-dioxygenase.

It is increasingly recognized that bidirectional communication exists between the gastro-intestinal tract and the central nervous system (CNS, Figure 2) (113). Different regions in the CNS such as the amygdala, hippocampus, and prefrontal cortex are involved both in the modulation of gut function as well as in the regulation of emotional and cognitive behaviors (114). Different anatomical sites determine gut-brain interaction including the CNS, the autonomic nervous system (ANS), the enteric nervous system (ENS), and the hypothalamus-pituitary–adrenal (HPA axis); while immune, endocrine, and neuronal mediators control these interactions (115). Another key player in the gut-brain axis is the gut microbiome which can influence gut-brain communication in different ways, interacting with all pathways implied in the gut-brain axis, including local interaction with the ENS and direct communication with the CNS through neuroendocrine and metabolic pathways (115, 116). Importantly, the CNS is an immunologically privileged site, maintained by three different barriers: the blood–brain barrier (BBB), the blood cerebrospinal fluid barrier (BCSFB), and the arachnoid barrier, which in physiological conditions limits the entrance of immune cells into the brain (117). Whereas the BBB is an endothelial barrier that strictly blocks the entrance of leukocytes into the brain (117), the BCSFB is formed by the choroid plexus which is a highly vascularized structure present in all brain ventricles that plays an important role in immune surveillance and regulation of immune cell trafficking (118).

### 4.1 Metabolic and microbial pathways

#### 4.1.1 Tryptophan metabolism and fatigue

Tryptophan (Trp) is an essential amino acid that is metabolized along three different pathways (119). The kynurenine pathway represents the main Trp degradation route, in which Trp is metabolized to kynurenine by indoleamine 2,3-dioxygenase 1 (IDO1) or tryptophan 2,3-dioxygenase (TDO) (120). Whereas TDO predominantly mediates the basal metabolism of kynurenine, IDO1 is induced by inflammatory cytokines at the transcriptional level (121), which is also observed in patients with active IBD, thus shifting Trp metabolism toward the kynurenine pathway (120, 121). Even when disease remission is reached, *Ido1* expression has been shown to remain high (122). Both Trp and kynurenine can pass the BBB, after which enzymes expressed in astrocytes and microglia can lead to the formation of neuroactive kynurenine metabolites in the CNS. These are categorized as excitotoxic (including 3-hydroxy-kynurenine and quinolinic acid) and neuroprotective (including kynurenic acid) (121, 123). The kynurenine 3-monooxygenase enzyme (KMO), leading to formation of 3-hydroxy-kynurenine, is mainly expressed in immunocompetent cells and the excitotoxic kynurenine pathway which is only weakly active under normal circumstances, is strongly induced during inflammatory conditions (123). Kynurenic acid is generated from kynurenine through the action of various kynurenine aminotransferases. These enzymes exhibit a lower affinity for kynurenine compared to KMO and increased activity of KMO further shifts the metabolic pathway toward the production of neurotoxic metabolites (124). In patients with depression, without antidepressant treatment, lower levels of kynurenic acid and higher levels of quinolinic acid were seen in the blood (125).

The second Trp pathway is the serotonin pathway, in which tryptophan hydroxylase (TPH) leads to the conversion of Trp to 5-hydroxytryptophan (5-HTP), the direct precursor of serotonin, a well-known neurotransmitter associated with the regulation of mood, behavior, and cognitive function (126–128). Both Trp as well as 5-HTP can cross the BBB, in contrast to serotonin itself (126).

In a third Trp pathway, intestinal bacteria process Trp to indoles, some of which can act as ligands for the aryl hydrocarbon receptor (AhR) (120). The latter plays an important role in the intestinal immune system, and mainly exerts anti-inflammatory effects in the gut through IL-22 (120). Again, inflammation induced upregulation of *Ido1* can impede the formation of AhR ligands, and an inflammation-induced downregulation of AhR has been described in patients with CD (120, 121). Besides the metabolization of Trp, the microbiome can also influence the kynurenine and serotonin pathway by increasing IDO1 and TPH1 activity (120, 126). Interestingly, a variety of bacteria express tryptophanases and other enzymes in the Trp pathway that will theoretically further influence the fate of dietary Trp in the colon (129), but detailed studies in that area are lacking.

Reduced serum Trp levels have been associated with fatigue in patients with cancer (130), chronic renal insufficiency (131), and in stroke survivors (132). In patients with IBS, fatigue was significantly associated with a polymorphism in *Tph2*, supporting a role for dysregulated serotonin synthesis in fatigue pathophysiology (133). In patients with IBD, reduced serum Trp levels have consistently been linked to active disease (134, 135). The evolution of serum Trp levels during stages of disease remission is more ambiguous. In the study of Gupta et al. in four patients with CD in remission, Trp levels were comparable to the healthy controls (135). In a larger study cohort of Nikolaus et al., inactive CD was associated with lower Trp levels compared to the healthy controls (134). Recently, Tran et al. followed a cohort of 76 patients throughout the induction phase of biological treatment and found a strong correlation between Trp levels and FACIT-F scores (29). Noteworthy, the FACIT-F correlated strongly with the disease activity, and an influence of disease activity both on fatigue as well as on Trp levels could also impact this relationship. Nevertheless, in a study assessing metabolite profiles in IBD in remission, patients with fatigue had lower serum Trp levels compared with their non-fatigued counterparts (43).

#### 4.1.2 The microbiome composition

The microbiome continuously interacts with the host’s epithelial and immune cells, ensuring homeostasis (136). Intestinal bacteria actively metabolize unabsorbed carbohydrates, exfoliated epithelial cells, and mucus, which in turn produces multiple metabolites such as short chain fatty-acids (SCFAs), that influences the host’s energy balance, immune system, and intestinal epithelial function (120, 136). The microbiome also regulates microglial homeostasis by the production of SCFAs (137) and these SCFAs have shown the potential to reduce lipopolysaccharide (LPS)-induced microglial activation *in vitro* (138).

Multiple studies in germ-free mice have confirmed the importance of the microbiome in different aspects of physiology, including normal functioning of the gut-brain axis (139, 140). Disruptions in the microbial equilibrium, termed dysbiosis, can render the gut vulnerable to pathogenic insults (141). In case of IBD, several studies have identified an overall reduction in the diversity of the microbiome, a reduction in the abundance of anti-inflammatory taxa, and a decrease in beneficial SCFA-producing bacteria (136, 142). Both depression and IBD separately have been linked to dysbiosis, but recently evidence also suggested differences in bacterial communities in patients with IBD and depressive symptoms compared to IBD patients without those symptoms (143). Additionally, fecal microbiome transplant from patients with IBD and depression to healthy mice induces not only colitis, but also depression-like behaviors, an effect that was not seen when transplanting the microbiome of patients with IBD without depression (144).

Previously, markers of gut dysbiosis, including circulating levels of LPS and antibodies against bacterial endotoxins, were associated with the severity of the chronic fatigue syndrome (145). Interestingly, the microbiome also has a crucial role in the motivation for exercise, as mice treated with broad-spectrum antibiotics show a decreased exercise performance (146). One study in patients with IBD showed that both fatigue and depression were associated with a distinct microbiota composition including lower abundances of SCFA-producing bacteria (147). Additionally, fatigue in patients with IBD in clinical and endoscopical remission was linked to a decrease in the diversity of the gut microbiome and reductions in butyrate-producing bacteria (43).

### 4.2 Immune pathways and neuroinflammation

Increasing evidence suggests that damage to the gut barrier integrity can lead to translocation of bacterial compounds across the gut epithelium, affecting the function of the BBB (148). Acute colitis in mice can lead to a decrease in the expression of tight junction proteins at the BBB, persisting up to 21 days after DSS initiation, suggesting a sustained effect of colitis (149, 150). Besides the BBB, the BCSFB barrier can also be severely affected by systemic inflammatory diseases, leading to increased permeability or alterations in the transcriptome of the choroid plexus (151–153).

The relationship between gut inflammation and neuroinflammation was shown in different animal models of acute and chronic colitis. In the brain of mice with acute colitis, RNA sequencing data revealed enriched inflammation-related pathways including the IL-17 signaling pathway, regulation of inflammatory responses, and antimicrobial peptides pathway (154). Differentially expressed genes included lipocalin-2 (*Lcn2*), *S100a8*, and *S100a9* (154). The latter two genes encode for proteins that form the heterodimer calprotectin (S100A8/A9), a well-known disease marker in IBD (155). Lipocalin-2 has a critical involvement in the maintenance of intestinal homeostasis, and both serum and fecal Lcn2 levels correlate with disease activity in patients with IBD (156–158). Acute colitis, induced by DSS or DNBS, was also associated with increased expression of inflammatory genes, such as *Tnf-α* and *Il-6*, in the hippocampus (154, 159, 160). Soon after the induction of colitis through DSS administration, an effect on the microglial phenotype has been noted, including a shortening of branch length and decrease in the number of junctions (152). Moreover, acute DSS-induced colitis has been associated with increased Iba1 immunoreactivity (154), a marker of both resting as well as activated microglia. In mice, acute colitis has also been associated with behavioral alterations including increased anxiety-like behavior and a decrease in general locomotion (161). Moreover, acute colitis resulted in a reduction of voluntary wheel running behavior, which is a measure of general activity, potentially indicating fatigue-like behavior (162).

Studies in mice with active chronic colitis confirmed that chronic gut inflammation is associated with sustained neuroinflammatory responses. Mice with chronic colitis showed reduced hippocampal brain activity, astrocytosis and astrocyte activation, and an upregulation of inflammatory cytokines, such as IL-6, TNF-α, and IL-1β, in the hippocampus (163–165). This increase in inflammatory mediators in the hippocampus during chronic DSS-colitis was associated with reduced long-term memory (163). Additionally, chronic DSS-induced colitis has been linked to an increase in microglial and astrocytic density, both in the hippocampus as well as in the cortex (166). Salvo et al. found an increase in hippocampal microglia following low-dose DSS in early life, leading to decreased neurogenesis in the hippocampus, ultimately resulting in behavioral deficits (167). Chronic experimental colitis has also been linked to significant neuronal loss and reduced neurogenesis in the hippocampus, suggesting a role for colitis in neurodegeneration (164, 166).

He et al. found that chronic colitis leads to a significant upregulation of the NLR domain-containing protein 3 (NLRP3) inflammasome, both in microglia as in astrocytes and was associated with enhanced anxiety-like behavior and signs of cognitive dysfunction (166). Recent evidence suggests an interaction between the NLRP3 inflammasome and the gut microbiome, which shapes the peripheral and central immune responses (168). Dysbiosis and changes in the enteric, peripheral, and central activation of the NLRP3 inflammasome were previously identified in patients with Parkinson’s disease, Alzheimer’s disease, and depression (168). In these diseases, overactivation of the NLRP3 inflammasome is thought to impair the BBB and contribute to the central inflammatory response, contributing to CNS pathology (168).

Different pathways can convey the inflammatory response from gut to the brain, including an increase in circulating levels of inflammatory mediators such as IL-6, CXCL-1, and HMGB1 as identified in different animal studies (149, 150, 154, 163). These inflammatory mediators can signal to the brain either through neuronal pathways or through direct access into the CNS (149). Additionally, the increased permeability of the CNS barriers can lead to translocation of immune cells into the brain further propagating neuroinflammation (155). In mice with acute colitis, an increase in the rolling and adherent leukocytes on cerebral endothelial cells was seen, which can direct recruitment of neutrophils into the brain vasculature (169). Likewise, acute colitis was associated with an increase in monocytes and neutrophils in the brain of mice (154). In a model of chronic colitis, the adoptive T-cell transfer model, an increase in BBB permeability and brain-infiltrating T-cells was noted (165).

### 4.3 Neuronal pathways

The ANS consists of parasympathetic (including the vagus nerve) and sympathetic nervous systems, which relays information from the ENS to the CNS (170). The vagal nerve has anti-inflammatory effects in the gut by the release of acetylcholine, which inhibits pro-inflammatory cytokine secretion by macrophages (171). This cholinergic anti-inflammatory pathway (CAIP) can be stimulated or inhibited by vagal nerve stimulation (VNS) or vagotomy, respectively. Vagotomy in mice with experimental colitis worsens disease activity (172), while VNS improved stool quality and reduced inflammation in rats with colitis (173). Moreover, both the prefrontal cortex and hippocampus hold modulatory roles over vagal nerve functioning, and it is hypothesized colitis-induced neuroinflammation might trigger vagus nerve dysfunction (155). Interestingly, patients with IBD and positive affective adjustment (lower scores of anxiety and depression) had more beneficial coping strategies and an adapted ANS activity (114). In patients with CD and positive affectivity, higher sympathetic activity was observed, probably indicating an adaptation mechanism since the sympathetic nervous system is known to exert anti-inflammatory effects in CD (114). In patients with UC, negative affectivity was associated with a parasympathetic blunt (114). Thus, ANS dysfunction could also play a role in IBD-related fatigue, but further research into this topic is required.

### 4.4 Endocrine pathways

Stress is a universal aspect of life, and the HPA axis plays a crucial role in our ability to adapt and respond to stressors, including the increase in blood sugar and suppression of the immune function (174).

Activation of the HPA axis plays a crucial role in the communication between immune and neuroendocrine systems, which is important in regaining homeostasis after immune activation (151). In patients with IBD, chronic exposure to stress and inflammation can result in dysregulation of the HPA axis (155, 175). This is supported by evidence correlating serum cortisol levels with psychological stress in CD (175). In healthy adults, the ANS and HPA axis cooperate in the anti-inflammatory response to stress, whereas these appeared to be uncoupled in patients with IBD (176, 177). In mice, acute DSS-induced colitis was linked to alterations in the HPA axis activity resulting in an altered behavioral response to stress (178). Moreover, induction of stress in mice led to activation of IL-6 signaling which was reported to play an important role in the changes occurring in the HPA axis in response to repeated stress (179). Chronic stress also induces visceral pain in mice, a process mediated by microglia activity in the central nucleus of the amygdala (180). Potentially, inflammation in the gut feeds forward to the CNS, leading to neuroinflammation-induced damage to brain regions implied in HPA axis regulation (155). In addition, the HPA axis can also influence behavior through modulation of the Trp metabolism since cortisol is known to activate TDO, leading to increased kynurenine production, and increased serotonin re-uptake, resulting in lower availability of serotonin which could enhance depressive symptoms (155). Patients with IBD often require courses of corticosteroids due to disease flare-ups, which can also impact the function of the HPA axis and lead to adrenal insufficiency, which can be quite prolonged (181, 182). Symptoms of adrenal insufficiency are rather non-specific but can include fatigue (181). Interestingly, patients with multiple sclerosis and fatigue had a higher activity of the HPA axis, with elevated plasma levels of ACTH, compared to their non-fatigued counterparts (183). On the contrary, in patients with IBD in remission without steroid use in the last year, no correlation was found between cortisol levels and fatigue (184). Thus, despite the apparent dysregulation of the HPA-axis in IBD, evidence on a role in fatigue pathogenesis remains limited.

### 4.5 Structural and functional alterations in the CNS of patients with IBD

As seen in other neurodegenerative diseases, several MRI studies have shown both structural and functional alterations in the CNS of patients with CD (185–187). Patients with CD had lower glutamate and glutamine concentrations in the brain, which was previously seen in Alzheimer’s disease (188). Additionally, in patients with CD, the volume of the choroid plexus was negatively correlated with biochemical markers of disease activity (CRP and fecal calprotectin) (185). These findings support the potential impact of systemic inflammation leading to alterations in the CNS, which could contribute to fatigue pathogenesis (188).

As mentioned earlier, IBS is linked to tiredness associated with IBD and among patients with CD in remission, whether experiencing abdominal pain or not, variations were observed in the resting-state brain activity within specific brain regions (189). Moreover, patients with CD exhibit changes in gray matter volume and cortical thickness compared to healthy controls, although when adjusting for anxiety and depression, the alterations in various brain regions implicated in emotional processing lose significance (187). Fatigue in patients with CD correlates with diminished gray matter volume in the precentral gyrus and other sensorimotor areas, particularly evident during periods of disease remission (190). Furthermore, significant disparities in cognitive functioning, cerebral perfusion, and neurochemistry are observed in patients with quiescent CD experiencing fatigue, compared to their healthy counterparts (188).

## 5 The management of IBD-related fatigue

**Figure 3:**
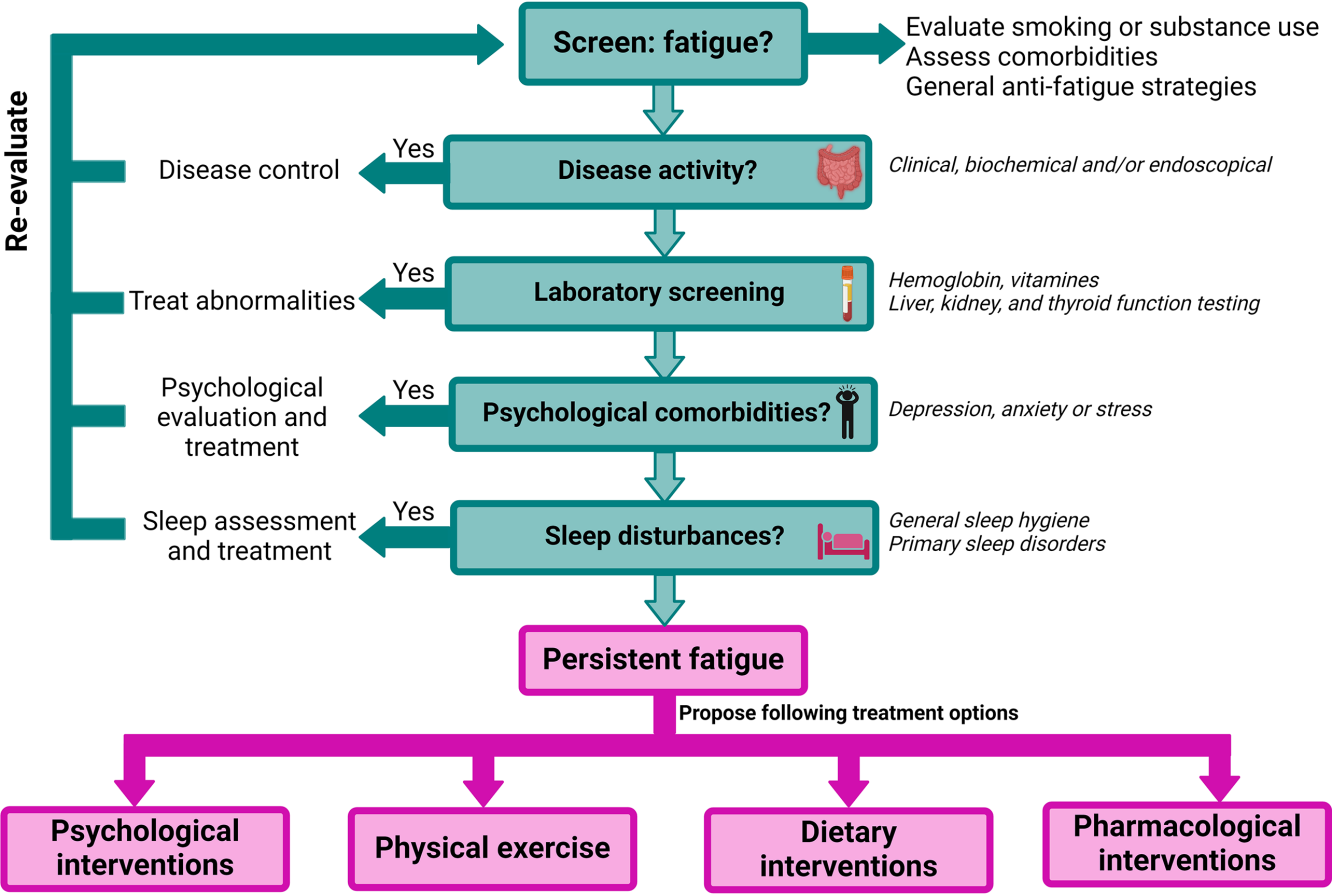
Proposed flowchart for the management of fatigue in IBD, adapted from Hindryckx et al. (21), Nocerino et al. (22), and Borren et al. (192). Figure created with BioRender.com.

Despite the high prevalence of fatigue, effective evidence-based treatment options remain limited. In general, health-care professionals’ experience multiple barriers in assessing and treating fatigue due to difficulties in conceptualizing this subjective complaint, the limited understanding of the impact of fatigue on the patients’ lives and/or gaps in their knowledge of the pathogenesis (191). Furthermore, patients sometimes feel their fatigue complaint is either ignored or they themselves do not report it to their physician since they are unaware it can be a manifestation of IBD (191). Therefore, the first step in the management should be to raise awareness, screen, and acknowledge patients’ fatigue levels (Figure 3). Additionally, general strategies can be proposed (193). This includes day planning, structured rests and breaks, distribution of their energy throughout the day and prioritization of important events (193). Maladaptive coping strategies such as daytime naps and all-or-nothing behavior should be discouraged (65, 194) and support from relatives can be advantageous in accepting and dealing with fatigue (193). To optimize communication with the physician during consultations patients can be encouraged to keep a fatigue diary, as this was proven beneficial in cancer-related fatigue (195, 196). In the future, there might also be a place for the use of telemedicine tools to offer patients continuous and personalized monitoring of the disease and its complications (including fatigue), such as the recently developed tool myIBDcoach® (197).

### 5.1 Treating active inflammation

Given the well-known association between fatigue and disease activity management of IBD activity is essential. Part of this treatment optimization is the assessment of treatment adherence as psychiatric comorbidities have been associated with noncompliance (198). Several studies have shown a beneficial effect of biologicals on fatigue and QoL and increasingly, fatigue is being incorporated as a formal outcome in randomized controlled trials (RCTs) assessing newer treatments for IBD. For example, TNF-inhibitors (199, 200), JAK-inhibitors (201, 202), and IL23-inhibitors (203–205) have shown beneficial effects on fatigue in patients with IBD. Moreover, some head-to-head trials have assessed advanced therapies for addressing fatigue associated with IBD. In patients with CD, ustekinumab demonstrated superior efficacy in reducing fatigue compared to adalimumab (206), while vedolizumab and tofacitinib showed similar effectiveness in managing fatigue among patients with UC (207). Despite these promising results, fatigue often persists during disease remission (208) and no data exist on the efficacy of biologicals for the treatment of fatigue in patients with clinically inactive IBD (192).

### 5.2 Correction of anemia and nutritional deficiencies

In patients with IBD and low hemoglobin levels, further anemia workup should be initiated, including iron and vitamin studies and substitution is recommended in case of deficiencies (209). In patients with IBD and iron-deficiency anemia, both ferric carboxymaltose (FCM) and ferric derisomaltose (FDI) led to a significant improvement in FACIT-F scores, with the greatest improvement in the FDI group (210). Treatment with FCM led to higher rates of hypophosphatemia and the severity of post-infusion hypophosphatemia was associated with slower improvement in fatigue (210). Evidence is less clear when assessing the need for iron supplementation in non-anemic patients with IBD-related fatigue. On the one hand, in the study of Ҫekiç et al., patients with IBD, all non-anemic with iron deficiency, showed a significant increase in QoL after an intravenous (IV) infusion with iron sucrose (211). On the other hand, in the RCT performed by Fiorino et al., IV FCM did not improve chronic fatigue in IBD patients with non-anemic iron deficiency (212).

There is even less evidence for a beneficial effect of vitamin substitution on IBD-related fatigue. In an RCT including 214 patients with IBD (without vitamin deficiencies), supplementation with an over-the-counter multivitamin and mineral supplement led to some alleviation of fatigue, particularly noticeable in patients with UC (213). Another RCT in 95 fatigued patients with IBD or IBS, and normal vitamin B_12_ levels, could not identify a beneficial effect of additional vitamin B_12_ supplementation (1,000 μg per day) on fatigue levels (214). Vitamin D substitution has been shown to be effective to reduce fatigue in patients with other IMIDs such as systemic lupus erythematosus (215) and to reduce health-care utilization (56) and improve health-related QoL (216) in patients with IBD. Nonetheless, there is insufficient evidence supporting vitamin D treatment for IBD-related fatigue.

### 5.3 Identification and treatment of mood and sleep disorders

In a next step, psychological comorbidities should be assessed since these are not only strongly related to fatigue but also to other adverse IBD-related outcomes (9, 10). In case of clinical suspicion, patients should either be treated by their IBD specialist or be referred to mental health services for further evaluation and treatment (217).

A routine evaluation of sleep quality should be performed in patients with IBD, especially in case of fatigue (72). When poor sleep quality is suspected, known risk factors for sleep disturbances such as mood disorders, medication use or smoking should first be identified and treated (72). Patients should be counseled on the importance of adequate sleep hygiene and aim to reach at least 7 h of sleep per night (218). Up to date, melatonin supplements, to improve the circadian function, cannot be recommended as insufficient evidence is available on their safety and effectiveness in patients with IBD (218). In case of unexplained poor sleep quality, a thorough assessment by a sleep professional may be recommended to exclude primary sleep disorders (72).

### 5.4 Treatment of persisting fatigue: non-pharmacological interventions

When other potential causes of fatigue are excluded and/or treated, re-evaluation should be performed, and potentially modifiable risk factors should be treated. In case of persisting, unexplained fatigue, psychosocial, physical or pharmacological interventions can be considered.

#### 5.4.1 Psychosocial interventions

Several trials have assessed psychological interventions in IBD, with fatigue either as a primary or secondary outcome. Different psychological interventions were tested including stress management techniques (219), solution-focused therapy (220, 221), cognitive behavioral therapy (222), mindfulness-based cognitive therapy (223), and psychoeducational sessions (224). Globally, all trials showed a beneficial effect on fatigue, even though this could not always be confirmed statistically, supporting a promising role for psychological therapies in the management of fatigue in quiescent disease (225). In contrast with the other trials, the study of Hashash et al. specifically included patients with reduced sleep quality, and in a first phase, behavioral therapy showed beneficial effects on both sleep and fatigue (226). Whereas the majority of other trials included patients in remission, a recent study assessed the effect of a cognitive behavioral and mindfulness-based stress reduction (COBMINDEX) program in patients with mild to moderately active CD (Harvey Bradshaw Index of 5–16) (227). After 3 months of intervention, patients in the COBMINDEX group experienced a significant reduction of their fatigue levels compared to baseline (227).

Notwithstanding, due to the scarce number of trials, heterogeneous study designs, underpowering, and small effect sizes, it is difficult to draw definite conclusions as regards to the most recommendable intervention in patients with IBD-related fatigue (225). In addition, little is known about the long-term impact of these interventions. For example, in the study of Vogelaar et al. the effect of solution-focused therapy disappeared at 9 months of follow-up (221). Some patients with IBD also experience barriers to engage in psychotherapy, which can severely impact the success of these interventions (228). For example, in adolescents and young adults with IBD and depression, patients identified fatigue as an important barrier to participate in mindfulness (229).

#### 5.4.2 Physical activity

Physical activity has numerous beneficial effects such as increasing anti-inflammatory adipokines, increasing bacterial diversity, and decreasing visceral adiposity (230). In addition, exercise is thought to improve well-being, stress, and QoL of IBD patients and might play a role in disease management (83). There is some evidence for a positive effect of physical exercise on fatigue levels, although fatigue was rarely the primary outcome measure, and several studies did not include a control group. In a pilot trial including 52 patients with CD individual exercise advice significantly improved fatigue, as measured with the IBD-F, one of the secondary outcomes, as compared to placebo (231). Fagan et al. evaluated the use of an unsupervised exercise program (an individualized instruction booklet) in patients that were either in remission or had mild to moderate disease activity, which led to a significant improvement in fatigue and QoL (232). In another cohort of 20 patients with CD, low intensity exercise improved QoL and even though fatigue was not an outcome measure in this study, patients did report increased energy levels and complained less about fatigue (233). The PROTECT trial assessed the effect of a 6-month exercise program, consisting of both impact as well as resistance training. Whereas fatigue was not a primary outcome of this trial, patients in the exercise group did show significantly lower fatigue scores compared to the controls (234). In a small case series of Nathan et al., patients with IBD who performed regular exercise were asked about benefits and difficulties they experienced, and four out of 11 patients felt that exercise helped them with fatigue (235). Another pilot trial in 32 patients with IBD found that a 16-week exercise program (IBD-Fit), significantly improved patients’ physical fitness and fatigue scores (236). In a larger survey assessing exercise habits of 918 patients with IBD, up to 12% felt physical activity helped them boost their energy levels (90). On the other hand, in a pilot study assessing the effect of high intensity interval training and moderate intensity continuous training, no clear effect was seen of these exercise regimens on fatigue levels (237). A potential benefit of yoga-based interventions can also be expected as these have previously been shown to improve QoL of patients with IBD and fatigue in patients with cancer and multiple sclerosis. However, trials in patients with IBD-related fatigue are currently lacking (238).

More research is required with fatigue as a primary outcome measure in physical activity intervention studies. Nevertheless, a beneficial trend is seen, and exercise interventions were generally well-tolerated and safe. Thus, for patients medically fit to do so, increasing rather than reducing exercise should be considered to address fatigue. During IBD consultations the international Physical Activity Questionnaire (IPAQ) can be used to assess patients current physical activity levels and discuss potential for improvement (84, 239).

#### 5.4.3 Diet

The high prevalence of IBD in Western countries underscores the importance of diet and its impact on the development of IBD (240). Given the associations between vitamin deficiencies, sarcopenia, and an unhealthy diet with fatigue, dietary interventions also hold promise for managing IBD-related fatigue, although up to date studies are still limited and often uncontrolled.

In a study with 26 patients with mildly active IBD or in IBD remission, diet quality was improved by increasing the intake of vegetables, fruits, whole grains, legumes, nuts, dairy, and fish and decreasing consumption of red and processed meat, sweetened beverages, alcohol, and unhealthy choices. This improved diet was significantly associated with reduced fatigue (241). Additionally, another intervention program consisting of one-on-one counseling sessions with a dietitian, resulted in improvement of fatigue in up to 94% of patients (242). Strobel et al. examined a holistic functional medicine approach encompassing sessions on nutrition, lifestyle and sleep. Additionally, patients were started on a six-week elimination diet, avoiding dairy, eggs, gluten, peanuts, shellfish, beef/red meat, soy, corn, refined sugar, caffeine and alcohol, followed by a calculated food reintroduction. In this first pilot trial, a significant improvement of fatigue was noted (243). A diet low in Fermentable oligosaccharides, disaccharides, monosaccharides and polyols (FODMAPs) is currently often used in patients with IBS, in whom it has been shown to ameliorate gastrointestinal symptoms, fatigue and QoL (244, 245). A recent systematic review assessed the effectiveness of a diet low in FODMAPs in patients with IBD and even though the diet was not effective in reducing stool consistency or mucosal inflammation, a significant reduction in fatigue levels was seen (245).

To conclude, dietary interventions can improve symptoms of fatigue, but further confirmation is required through larger randomized controlled trials. Of note, caution is needed when imposing a restrictive diet, such as the FODMAP diet, on patients with IBD due to the high prevalence of malnutrition. These diets should be supervised by a dietitian (246).

#### 5.4.4 Other non-pharmacological interventions

Some anecdotical evidence exists for other interventions such as (electro)acupuncture (247), a mushroom supplement (AndoSan™) (248), and aromatherapy (249) in the treatment of IBD-related fatigue. Other natural supplements such as omega-3 showed no effect on fatigue (231). Since the evidence from these smaller, single-center studies is rather limited, further studies are required to confirm the effectiveness of these interventions for the treatment of IBD-related fatigue.

### 5.5 Treatment of persisting fatigue: pharmacological interventions

#### 5.5.1 Thiamine

In an initial open label trial high-dose thiamine led to complete regression of IBD related fatigue (250), which was subsequently confirmed in a cross-over RCT assessing the effectiveness of high-dose oral thiamine (dose depending on the sex and body weight) for 4 weeks (251). Treatment with thiamine led to a significant reduction of fatigue as measured with the IBD-F compared to placebo after which an increase of fatigue burden was seen (251). Noteworthy, thiamine treatment had no impact on handgrip strength, one of the objective markers of fatigue. Next, a long-term extension trial was performed to assess the effect of continued treatment with low dose thiamine (300 mg for 12 weeks), however, this trial could not show a beneficial effect over placebo (252). Therefore, even though a short thiamine course can be proposed, no evidence exists on how to proceed after this treatment.

#### 5.5.2 Antidepressants

Some studies have also assessed the effect of antidepressants on patients with IBD without any evidence for an uncontrolled depressive or anxiety disorder. In this setting, the effect of antidepressants on QoL is rather conflicting, with a beneficial effect of duloxetine (253) over placebo, but not with fluoxetine (254). A recent trial investigated the effect of brief behavioral therapy for sleep in IBD (BBTS-I) with or without the antidepressant bupropion on fatigue in patients with IBD with poor sleep quality (226). Even though in both groups a significant improvement of fatigue was seen, bupropion + BBTS-I was not better than BBTS-I alone (226).

#### 5.5.3 Other pharmacological interventions

Methylphenidate is a psychostimulant that increases dopamine levels in the CNS and is mainly used in the treatment of attention deficit disorder (255). Some data suggest a therapeutic effect of methylphenidate on cancer related fatigue, yet information on the effectiveness, safety and long-term risks is still limited (255). Additionally, methylphenidate has not been studied in the context of IBD-related fatigue, thus cannot be recommended in this setting.

### 5.6 Modulation of the gut-brain axis

#### 5.6.1 Targeting metabolomic pathways

Based on the evidence of reduced serum Trp levels related to fatigue (29, 43), in theory, Trp substitution can be considered for the treatment of fatigue. A recent crossover RCT compared the effectiveness of 5-HTP to placebo for the treatment of fatigue in patients with IBD in remission (256). Despite a significant increase in serum 5-HTP and serotonin levels, oral 5-HTP did not modulate IBD-related fatigue better than placebo.

Another strategy aimed at modulating the gut-brain axis is the use of probiotics, which have shown to improve intestinal barrier function, increase bacterial diversity, and inhibit the growth of potentially pathogenic bacteria (142, 192). Modest evidence suggests a beneficial effect of probiotics on fatigue levels in patients with post-COVID fatigue (257) and patients with chronic fatigue syndrome (258). In mice with colitis, probiotics have shown efficacy in the treatment of depressive-like behaviors and neuroinflammation (259). The effect of probiotics on IBD-related fatigue has not yet been tested, however, currently a trial is ongoing that compares the effect of a probiotic mix with placebo on IBD-related fatigue (260).

#### 5.6.2 Treatment of neuroinflammation

Some studies have attempted to diminish colitis-induced neuroinflammation. For example, prophylactic treatment with an S100A9 inhibitor has been shown to reduce colitis-induced neuroinflammation (154) and NLRP3^−/−^ mice showed attenuated neuroinflammation and neurological dysfunction following DSS (166). Although treating neuroinflammation associated with IBD could be a highly specific and novel approach to targeting fatigue, identifying the precise nature of these interactions requires carefully considered experimental designs, since such treatments often also mitigate the severity of the experimental colitis itself (154, 166).

#### 5.6.3 Modulation of neuronal pathways

Vagal nerve stimulation has previously been shown to induce both anti-inflammatory and anti-depressive effects and some evidence exists for a beneficial effect of vagal nerve stimulation on fatigue associated with IMIDs (155, 261). In a small cohort of female patients with Sjögren’s syndrome, vagal nerve stimulation was effective in improving fatigue scores, however, no sham control group was included (262). Another study in patients with systemic lupus erythematosus, non-invasive vagal nerve stimulation was significantly better than the sham procedure in improving fatigue (263). A successful pilot trial was performed with 9 patients with CD where chronic vagal nerve stimulation resulted in decreased alpha frequency bands as measured by electroencephalography. This decrease in alpha power was correlated with decreased anxiety and clinical improvement, although it should be noted that causality could not be inferred (264). It remains to be elucidated if vagus nerve stimulation could be effective in IBD-related fatigue.

## 6 Conclusion

Patients with IBD often encounter persisting fatigue, impacting their QoL both during active and remission stages of the disease. While the pathogenesis of IBD-related fatigue remains incompletely unraveled, various risk factors have been identified, offering opportunities for targeted management strategies. In case of persisting, unexplained fatigue several therapeutic options are available, including pharmacological and non-pharmacological approaches. In recent years, emerging evidence highlights the role of the gut-brain axis in fatigue pathogenesis, yet further research is needed to elucidate the precise interplay between chronic gut inflammation and CNS dysfunction. Increased understanding in this area is crucial for the development of novel therapeutic interventions aimed at alleviating IBD-related fatigue.

## Author contributions

MT: Writing – review & editing, Writing – original draft, Visualization, Conceptualization. HL: Writing – review & editing, Writing – original draft, Visualization. MD: Writing – review & editing, Supervision. DL: Writing – review & editing, Supervision. TL: Writing – review & editing, Supervision.
